# Utility of Biomarkers in the Differential Diagnosis of Heart Failure in Older People: Findings from the Heart Failure in Care Homes (HFinCH) Diagnostic Accuracy Study

**DOI:** 10.1371/journal.pone.0053560

**Published:** 2013-01-11

**Authors:** James M. Mason, Helen C. Hancock, Helen Close, Jerry J. Murphy, Ahmet Fuat, Mark de Belder, Raj Singh, Andrew Teggert, Esther Wood, Gill Brennan, Nehal Hussain, Nitin Kumar, Novin Manshani, David Hodges, Douglas Wilson, A. Pali S. Hungin

**Affiliations:** 1 Durham Clinical Trials Unit, Durham University, Queen's Campus, Stockton-on-Tees, United Kingdom; 2 School of Medicine, Pharmacy and Health, Durham University, Queen's Campus, Stockton-on-Tees, United Kingdom; 3 Department of Cardiology, Darlington Memorial Hospital, Darlington, United Kingdom; 4 Department of Cardiology, The James Cook University Hospital, Middlesbrough, United Kingdom; 5 Department of Pathology, The James Cook University Hospital, Middlesbrough, United Kingdom; The University of Texas Health Science Center, United States of America

## Abstract

**Background:**

The performance of biomarkers for heart failure (HF) in older residents in long-term care is poorly understood and has not differentiated between left ventricular systolic dysfunction (LVSD) and HF with preserved ejection fraction (HFpEF).

**Methods:**

This is the first diagnostic accuracy study in this population to assess the differential diagnostic performance and acceptability of a range of biomarkers against a clinical diagnosis using portable echocardiography. A total of 405 residents, aged 65–100 years (mean 84.2), in 33 UK long-term care facilities were enrolled between April 2009 and June 2010.

**Results:**

For undifferentiated HF, BNP or NT-proBNP were adequate rule-out tests but would miss one in three cases (BNP: sensitivity 67%, NPV 86%, cut-off 115 pg/ml; NT-proBNP: sensitivity 62%, NPV 87%, cut-off 760 pg/ml). Using higher test cut-offs, both biomarkers were more adequate tests of LVSD, but would still miss one in four cases (BNP: sensitivity 76%, NPV 97%, cut-off 145 pg/ml; NT-proBNP: sensitivity 73%, NPV 97%, cut-off 1000 pg/ml). At these thresholds one third of subjects would test positive and require an echocardiogram. Applying a stricter ‘rule out’ threshold (sensitivity 90%), only one in 10 cases would be missed, but two thirds of subjects would require further investigation. Biomarkers were less useful for HFpEF (BNP: sensitivity 63%, specificity 61%, cut-off 110 pg/ml; NT-proBNP: sensitivity 68%, specificity 56%, cut-off 477 pg/ml). Novel biomarkers (Copeptin, MR-proADM, and MR-proANP) and common signs and symptoms had little diagnostic utility.

**Conclusions:**

No test, individually or in combination, adequately balanced case finding and rule-out for heart failure in this population; currently, in-situ echocardiography provides the only adequate diagnostic assessment.

**Trial Registration:**

Controlled-Trials.com ISRCTN19781227

## Introduction

Whilst early, accurate diagnosis and management of heart failure (HF) may substantially improve prognosis, there is evidence to suggest that HF is missed in up to half of cases [1]–[2]. Although echocardiography is the reference standard for the diagnosis of HF it does not always provide a definitive result, due to difficulties of imaging in individual patients, particularly in older, comorbid populations. Diagnosis ideally requires accurate, accessible, cost-effective and acceptable alternatives to echocardiography [3].

B-type natriuretic peptide (BNP) has been available as a routine laboratory test since 2003 [4], [5], however, there is wide variation in cut-off levels, and recognition that the optimal threshold may be age dependent [1], [4], [6]. A recent European study of nursing-home residents [1] (n = 150) suggested a cut-off for NT-proBNP of 450 pg/ml (sensitivity 71%, specificity 67%) and BNP 100 pg/ml (sensitivity 71%, specificity 70%). Other studies suggest cut-offs ranging from 93–450 pg/ml for NT-proBNP and 40–100 pg/ml for BNP [1], [7], [8]. Consequently clinical guidelines provide several thresholds where chronic HF is unlikely (BNP<100 pg/ml or NT-proBNP<400 pg/ml) or likely (BNP>400 pg/ml or NT-proBNP>2000 pg/ml), with a range of uncertainty between [6], [9]. Despite recommendations that natriuretic peptides should be part of HF diagnostic pathways, their clinical use is relatively limited [10]. Previous studies have not fully evaluated their clinical utility in older residents in long-term care and have not differentiated between types of HF despite the increased prevalence of HF with preserved ejection fraction (HFpEF) in this population [3], [11]–[12]. Current cut-offs (based exclusively on LVSD) may not enable appropriate rule-in or rule-out judgements.

A number of novel biomarkers have possible clinical utility in HF diagnosis [13]–[15]. These include mid-regional pro atrial natriuretic peptide (MR-proANP), mid-regional pro adrenomedullin (MR-proADM) and C-terminal provasopressin (Copeptin); each easily measured using commercially available assays. MR-proANP was evaluated positively in a European/US study (n = 1641) as a rule out test for HF using a cut-off value of 120 pmol/L (sensitivity 97%, spec 59.9%, PPV 56%, NPV 97%) [16]. MR-proADM has been evaluated in the 90-day survival prognosis of HF with a suggested cut-off value of 1.985 pmol/L (sensitivity 53%, specificity 76%) [16]. Copeptin has been investigated in patients with HF following acute myocardial infarction. A cut-off value of 25.9 pmol/L was established for predicting one-year mortality (sensitivity 68%, specificity 83%, PPV 40%, NPV 94%) [17]. In patients with coronary heart disease, raised high-sensitivity C-reactive protein (hs-CRP) is an independent risk marker for HF [18], [19]. Cut-off values for hs-CRP have been suggested which stratify risk into low (<1 mg/L), intermediate (1–3 mg/L), high (>3–10 mg/L) [20] and very high (>10 mg/L) [21] categories. Given the uncertain utility of routine biomarkers, further evaluation of these easily available, low cost biomarkers is needed in older, long-term care populations; this is the first study to do so. Our aim was to determine whether BNP, NT-pro-BNP, MR-proANP, MR-proADM, Copeptin and HsCRP could serve as biomarkers for the detection of LVSD and/or HFpEF in older people in long-term care and whether appropriate cut off values would differ from those for the general population.

## Methods

### Study Population

**Figure 1:**
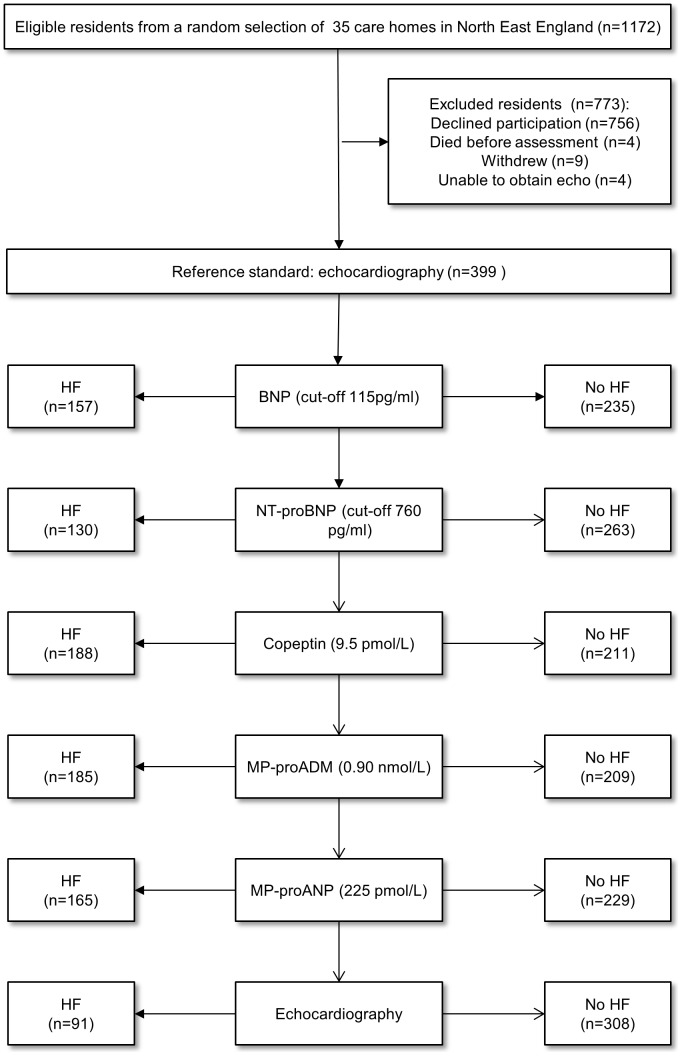
Flow diagram showing participation and biomarker findings for undifferentiated heart failure.

The cohort for this nested diagnostic accuracy study were participants in a prevalence study (see [22] for full details) and randomised controlled trial [23]. Between April 2009 and June 2010, 405 residents aged ≥65 years without terminal disease were recruited within 33 care homes in the North East of England. No exclusions were made on the basis of cognitive capacity, comorbidities or immobility. We extracted anonymised demographic details of all eligible residents (including non-participants) in order to assess their representativeness and thus the potential for selection bias (see [22] and Figure 1).

### Diagnostic Investigation

Each resident underwent a diagnostic assessment in their care facility, including Mini Mental State Examination [24], demographic details and past medical history, quality of life assessment using EuroQol: EQ-5D [25], electrocardiography, echocardiography, and blood tests. A doctor conducted the physical assessment including blood pressure, heart and respiratory rate, lung signs, displaced apex beat, third heart sound, jugular venous pressure, peripheral oedema, and New York Heart Association classification [26].

Portable echocardiograms (Vivid-i, application software version 6.2.0, system software 2.1.16, 3RS probe) were performed by a British Society of Echocardiography (BSE) accredited physiologist according to BSE guidelines [27], and electrocardiograms (ECGs; GE MAC 1600, cardiosoft version 6.5) by a trained phlebotomist. On completion of the study 50 echocardiograms were randomly selected and independently reported by an external BSE accredited cardiac physiologist; 100% agreement on LVSD status and valvular function was reached. Echocardiograms, ECGs and clinical assessments were conducted independently and mutually blinded.

### Presence or absence of heart failure

The clinical features, ECG and echocardiogram data were reviewed by two independent HF specialists (JJM+AF). A diagnosis of HF was made according to ESC guidelines [6], requiring objective evidence of a structural or functional abnormality of the heart at rest in the presence of appropriate symptoms and signs. Assessment of left ventricular systolic and diastolic function allowed sub-classification of LVSD and HFpEF [28], [29]. If symptoms and signs were absent but there was left ventricular systolic dysfunction, LVSD (without HF) was diagnosed [6].

Left ventricular ejection fraction was calculated by Simpson's rule and wall motion index using the American Society of Echocardiography 16 segment model [30]. All patients with clinical features and either a LVEF of ≤50% or whose left ventricular systolic function was assessed by ‘eyeball’ to be mildly, moderately or severely impaired, were classified as having HF due to LVSD. Doppler and tissue-doppler measurements of the longitudinal function of the heart were used to determine left ventricular diastolic dysfunction. E/E' measurements were recorded at both the septum and lateral wall. HFpEF was diagnosed in accordance with ESC guidelines [31] using clinical, echocardiographic and BNP measurements. Patients with clinical features of HF whose LVEF was >50% with E/E' >15, or those with an equivocal E/E' (8–15) but BNP >200 or NT-pro BNP >220 pg/ml were diagnosed as having HFpEF. Thus reference standard determinations of LVSD and HFpEF were clinically determined by interpreting echocardiography findings augmented with evidence from guidelines.

### Assays and sample processing

Blood samples for BNP were drawn into ethylenediaminetetraacetic acid-treated tubes, then centrifuged at 2,500 rpm for 10 min and stored at −20°C. Plasma BNP was measured in batches on a weekly basis with the Siemens Diagnostics Advia Centaur XP automated immunoassay analyser. The ADVIA Centaur BNP assay is traceable to an internal standard manufactured using synthetic human BNP (amino acid 77–108). The lower limit of detection (analytical sensitivity) for BNP was <2.0 pg/ml. The intra-assay coefficient of variation was 1.98–1.26% from 35.27–876.34 pg/ml.

Blood samples for NT-proBNP and hs-CRP were drawn into serum-separating tubes and for Copeptin, MR-proADM and MR-proANP into ethylenediaminetetraacetic specimen tubes. Samples were centrifuged at 3000 rpm for 10 minutes, serum and plasma aliquoted into secondary tubes 75×12 mm polypropylene tubes (Sarstedt, Leicester, UK) and stored at −80°C. NT-proBNP was assayed with the Roche Elecsys 2010 (Roche Diagnostics, Lewes, UK) using the Roche NT-proBNP II electrochemiluminescent sandwich assay. The analytical range was 5–35000 pg/ml; the inter-assay and intra-assay variabilities were 4.6–3.8% from 44–33606 pg/ml and 4.2–2.7% from 44–33606 pg/ml respectively. Hs-CRP was assayed on the Siemens Advia 2400 Chemistry analyser (Siemens Healthcare Diagnostics, Frimley, UK) using the Siemens wide range CRP latex-enhanced immunoturbidimetric assay. The analytical range for hs-CRP was 0.03–158 mg/L; the inter-assay and intra-assay variabilities were 4.9–7.8% from 2.25–49.96 mg/L and 3.2–5.2% from 2.25–49.96 mg/L respectively.

Copeptin, MR-proADM and MR-proANP were assayed on the Brahms Kryptor Compact analyser (Brahms UK Ltd, Bottisham, UK), utilising the Time Resolved Amplified Crytate Emission immunofluorescent assay principle. The analytical range for Copeptin was 4.8–500 pmol/L; the inter-assay and intra-assay variabilities were <15–<8% from 15–20 pmol/L and <17–<6% from 2–50 pmol/L respectively. The analytical range for MR-proADM was 0.05–10 nmol/L; the inter-assay and intra-assay variabilities were <20–6% from 0.2–6 nmol/L and <10–<3.5% from 0.2–6 nmol/L respectively. The analytical range for MR-proANP 2.1–1000 pmol/L; the inter-assay and intra-assay variabilities were ≤6.5% from 10–20 pmol/L and <4.5–<3.5% from 10–1000 pmol/L respectively.

### Data analysis

Descriptive statistics summarised participants' characteristics, HF status, and biomarker values. The proportion of correct diagnoses of LVSD and HFpEF in the biomarker groups were compared using Fisher's exact test. Other categorical variables, expressed as numbers and percentages, were compared using chi-square tests. Continuous variables were expressed as mean (standard deviation, range) and compared with an unpaired 2-sided Student t test when normally distributed. Normality was determined using the software program SPSS (version 19), using the Shapiro-Wilk test. Subgroup analysis explored echocardiography as the reference standard with and without clinical signs (including biomarkers) in the diagnosis of HFpEF.

Presence of clinically-assessed (echocardiographically-informed) LVSD and HFpEF were the principal end-points against which each diagnostic test was evaluated. Receiver operator curves were generated for each measure, with area under curve (AUC) estimates interpreted as a measure of the potential utility of each test, and test performance explored for optimal predictive values. ‘Optimal’ thresholds were determined as those providing the best balance of sensitivity and specificity to balance false positive and negative findings.

### Ethics statement

The study complies with the 1975 Declaration of Helsinki; the study received prior local research management and governance and national ethics approvals from Leeds West REC (Reference: 08/H1307/96). All participants provided informed consent prior to participation.

## Results

A total of 405 participants were screened, with a mean age of 84.2 y (SD 7.2, range 65–100 y). Of the total, 294 (74%) were female; all participants were white European; 393 (99%) were white British. There were four unsuccessful venepuncture attempts and six participants were excluded because of incomplete echocardiography data. A total of 399 participants were included in the final analysis. Study participants and non-participants showed similar baseline demographic characteristics (see [22]). Of 399 participants, 34 (8.5%) were diagnosed with LVSD: 19 (56%) mild, 9 (27%) moderate, and 6 (18%) severe; 3 with asymptomatic LVSD; 57 (14.3%) participants were diagnosed with HFpEF, of these 46 (81%) had E/E' >15.

### Individual blood test findings

BNP and NTproBNP levels were compared between patients with heart failure (LVSD or HFpEF) and those without. BNP values were lower in patients without HF (112 pg/ml, SD 121) than in patients with LVSD (406 pg/ml SD 413, p = <0.001), or in patients with HFpEF (161 pg/ml, SD 107, p = 0.003; see Table 1). NT-proBNP values were lower in those without HF (764 pg/ml, SD 1280) than in patients with LVSD (3910 pg/ml, SD 6065, p = 0.006), or HFpEF (1300 pg/ml, SD 1604, p = 0.020). Among patients diagnosed with LVSD, BNP and NTproBNP values were consistently and significantly associated with disease severity (BNP mean for mild: 270 pg/ml (p = <0.001), moderate 680 pg/ml (p = <0.001) and severe disease 428 pg/ml (p = <0.001); NTproBNP mean for mild: 2034 pg/ml (p = 0.037), moderate 8296 pg/ml (p = <0.001) and severe disease 3146 pg/ml (p = 0.034)).

**Table 1:** Findings of tests specific to heart failure (LVSD, HFPEF).

Blood test	No of residents	Normal range	Outside range		No HF (N = 308)			Undifferentiated HF (n = 91)					LVSD (n = 34)					HFPEF (n = 57)				
			N	%	Mean	Range	SD	Mean	Range	SD	AUC	(95% CI)	Mean	Range	SD	AUC	(95% CI)	Mean	Range	SD	AUC	(95% CI)
BNP	392	<100 pg/ml	183	47	112	6–1309	121	251	14–1797	287	0.73	(0.67–0.79)	406	30–1797	413	0.8	(0.71–0.89)	161	14–570	107	0.64	(0.56–0.71)
NT-proBNP	393	<400 pg/ml	214	55	764	30–14537	1280	2257	85–31249	4055	0.72	(0.66–0.78)	3910	130–31249	6065	0.78	(0.69–0.87)	1300	85–9611	1604	0.64	(0.57–0.71)
hs-CRP	394	0–5 mg/l	153	39	13	0.03–167	25	12	0.1–171	22	0.5	(0.43–0.57)	10	0.2–45	12	0.53	(0.43–0.63)	12	0.1–171	26	0.48	(0.40–0.56)
Copeptin	399	M: 0–19.1 pmol/L	109	37	16	5–184	20	21	4.7–154	29	0.56	(0.5–0.63)	20	5–71	18	0.59	(0.49–0.69)	22	5–154	34	0.53	(0.45–0.61)
		F: 0–12.9 pmol/L	34	32																		
MR-pro-ADM	394	0.23–0.55 nmol/L	352	89	0.95	0.08–8.78	0.62	1.2	0.3–3.9	0.6	0.67	(0.61–0.73)	1.26	0.30–3.92	0.82	0.6	(0.49–0.71)	1.18	0.55–2.79	0.44	0.68	(0.61–0.75)
MR-pro-ANP	394	7–85.2 pmol/L	369	94	225	47–969	139	338	71–929	204	0.69	(0.63–0.76)	391	87–929	254	0.68	(0.58–0.79)	308	71–916	165	0.66	(0.59–0.73)

Compared with patients without heart failure, novel biomarker levels for MR-proADM and MR-proANP were statistically significantly higher in both LVSD (p = 0.013, p = 0.001 respectively) and HFpEP (p = 0.022, p = 0.001 respectively) groups. Mean differences were non-significant for Copeptin for LVSD (p = 0.263) and HFpEF groups (p = 0.229).

Using AUC as a screening measure for test utility, BNP (AUC 0.80) and NT-proBNP (AUC 0.78) suggested potentially worthwhile tests for LVSD but not for HFpEF (see Table 1). Although there were significant differences in mean values for some of the other biomarkers evaluated, none had adequate potential utility as tests to diagnose LVSD or HFpEF in this population.

**Figure 2:**
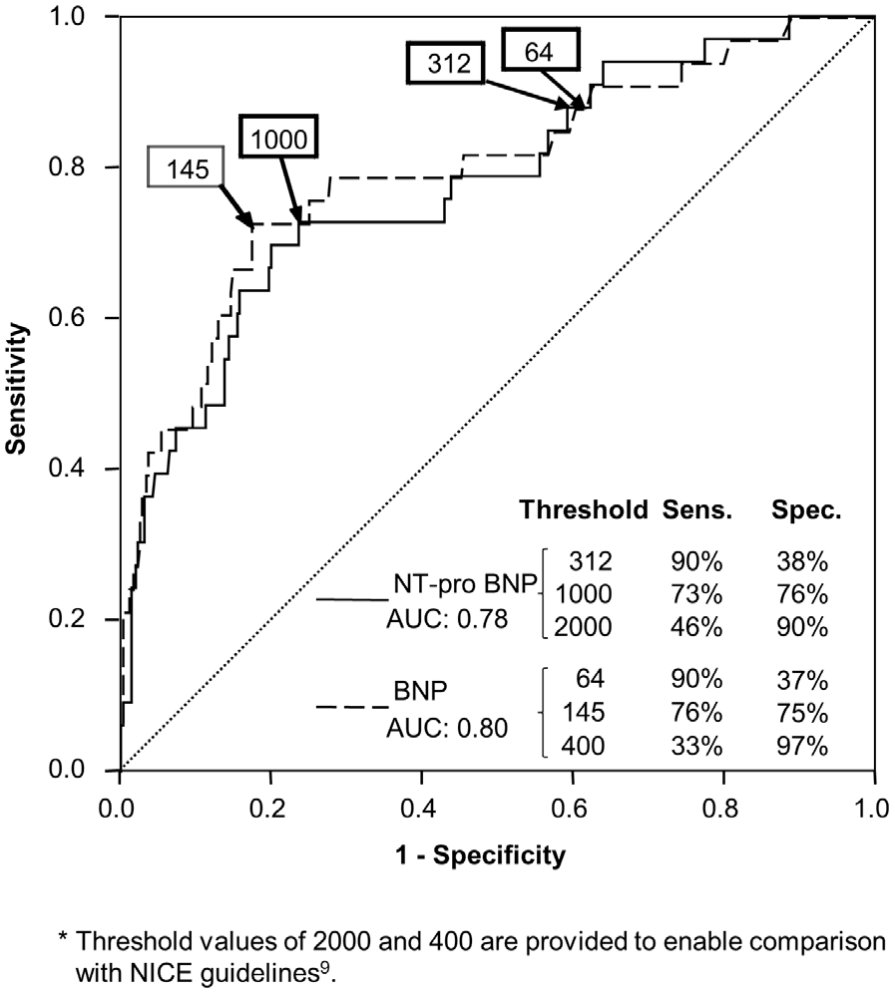
ROC curve: performance of NTproBNP and BNP in detecting LVSD.

The relationship between BNP and NT-proBNP threshold values and test accuracy in identifying LVSD (n = 34) is shown in Figure 2. A BNP threshold value of 145 pg/ml had sensitivity 76% and specificity 75% (PPV 22%, NPV 97%). A NT-proBNP value of 1000 pg/ml had sensitivity 73% and specificity 76% (PPV 22%, NPV 97%). For comparison, published general population cut-off values [9] are included (BNP: 400 pg/ml; NT-pro BNP: 2000 pg/ml), and miss more than half of cases of LVSD (sensitivity <50%).

For LVSD, with BNP at a cut-off of 145 pg/ml, 71% of participants would test negative and the test would have been correct (NPV) in 97% of these (see Table 2). Only 29% of subjects would have been selected for echocardiographic investigation. However, a test sensitivity of 76% means that one in four cases of LVSD would be missed; for our study sample, this cut off would have missed eight patients with LVSD, of whom six had mild, one moderate and one severe LVSD.

**Table 2:** Diagnostic test performance of diagnostic markers, signs and symptoms.

	Threshold value	Sensitivity	Specificity	Positive Predictive Value	Negative Predictive Value	Proportion testing Positive	Proportion testing Negative
**Either LVSD OR HFPEF**							
BNP	115 pg/ml	67%	68%	38%	88%	40%	60%
NT-proBNP	760 pg/ml	62%	75%	42%	87%	33%	67%
Copeptin	9.5 pmol/L	55%	54%	26%	80%	47%	53%
MR-proADM	0.90 nmol/L	67%	58%	32%	86%	47%	53%
MR-proANP	225 pmol/L	67%	64%	36%	87%	42%	58%
ECG	Abnormal†	70%	50%	29%	85%	54%	46%
Previous MI	-	17%	93%	42%	79%	10%	90%
BNP (no previous MI)	93 pg/ml	75%	60%	36%	89%	46%	54%
NT-proBNP (no previous MI)	488 pg/ml	71%	62%	36%	88%	44%	56%
MICE*	5 or above	56%	69%	35%	84%	20%	80%
	3 or above	81%	48%	32%	90%	37%	63%
**LVSD**							
BNP	145 pg/ml	76%	75%	22%	97%	29%	71%
NT-proBNP	1000 pg/ml	73%	76%	22%	97%	28%	72%
Copeptin	8.5 pmol/L	68%	51%	11%	95%	50%	50%
MR-proADM	0.88 nmol/L	64%	51%	11%	94%	50%	50%
MR-proANP	274 pmol/L	61%	73%	17%	95%	30%	70%
ECG	Abnormal†	85%	48%	13%	97%	54%	46%
Previous MI	-	24%	92%	22%	93%	10%	90%
BNP (no previous MI)	145 pg/ml	80%	76%	23%	98%	29%	71%
NT-proBNP (no previous MI)	1000 pg/ml	76%	77%	24%	97%	27%	73%
MICE*	5 or above	65%	66%	15%	95%	37%	63%
	3 or above	82%	43%	12%	96%	59%	41%
**HFPEF**							
BNP	110 pg/ml	63%	61%	13%	95%	42%	58%
NT-proBNP	477 pg/ml	68%	56%	13%	95%	48%	52%
Copeptin	10 pmol/L	53%	54%	10%	93%	47%	53%
MR-proADM	0.96 nmol/L	63%	61%	13%	95%	42%	58%
MR-proANP	218 pmol/L	70%	59%	14%	96%	45%	55%
ECG	Abnormal†	61%	47%	10%	93%	54%	46%
Previous MI	-	12%	91%	11%	92%	10%	90%
BNP (no previous MI)	110 pg/ml	62%	63%	13%	95%	40%	60%
NT-proBNP (no previous MI)	477 pg/ml	68%	58%	13%	95%	44%	56%
MICE*	5 or above	51%	65%	12%	93%	36%	64%
	3 or above	82%	45%	12%	96%	58%	42%

* Clinical scoring system to determine risk of heart failure (male = 2; infarction = 6; crepitations = 5; oedema = 3).

† As determined by a consultant cardiologist.

**Figure 3:**
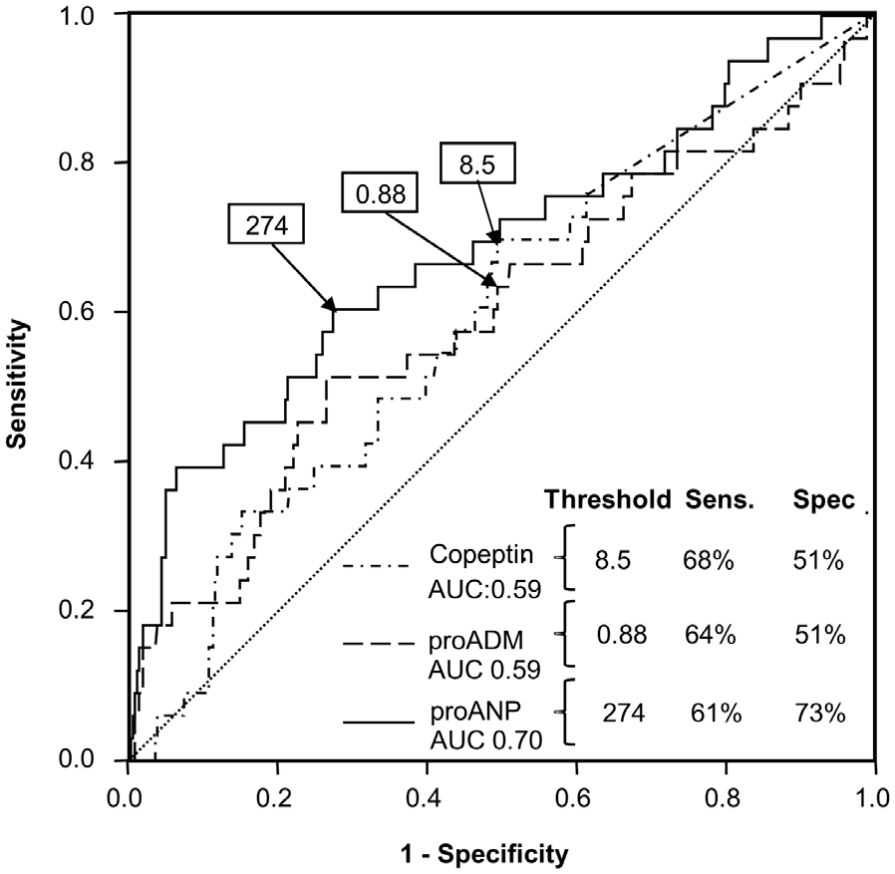
ROC curve: performance of novel assays in detecting LVSD.

The relationship between Copeptin, MR-proADM, and MR-proANP cut-off values and test accuracy in identifying LVSD is shown in Figure 3. Of these novel biomarkers, MR-proANP performed best, although not as well as BNP or NT-proBNP. A MR-proANP threshold value of 274 pmol/ml would rule out LVSD in 70% of subjects (with NPV = 95%) but miss 39% of cases (sensitivity 61%; see Table 2).

No biomarker provided an accurate test for HFpEF (n = 57) irrespective of E/E' diagnostic cut-off. For BNP and NT-proBNP optimal cut-offs were lower when screening for HFpEF compared to LVSD (see Table 2). A BNP cut-off of 110 pg/ml would reliably rule out HFpEF in 58% of subjects (with NPV 95%) but miss one in three cases (sensitivity 63%). Of the new biomarkers MP-proANP was most promising: at a cut-off of 218 pmol/ml, MP-proANP would reliably rule out HFpEF in 55% of subjects (NPV 96%) missing 30% of cases (sensitivity 70%).

For undifferentiated HF (LVSD or HFpEF, n = 91), BNP at a cut-off of 115 pg/ml, had a sensitivity of 67% and specificity 68% (PPV 38%, NPV 88%), and would have missed 30 cases (33%, see Table 2 and Figure 1). A NT-proBNP value of 760 pg/ml had sensitivity 62% and specificity 75% (PPV 42%, NPV 87%).

### Optimisation for ‘rule-out’

Test thresholds were re-evaluated for BNP and NTpro-BNP requiring 90% sensitivity in detecting LVSD. This was achieved at thresholds for BNP of 64 pg/ml and NT-pro BNP of 312 pg/ml (see Figure 2). For both biomarkers 36% of participants would have tested negative and the test would have been correct (NPV) in 98% of cases. At this threshold only one in 10 subjects with LVSD would have been missed, however 64% (n = 255) of subjects would have been referred for echocardiographic investigation.

### Individual clinical symptoms and signs

Common symptoms and signs associated with HF were evaluated; most of these performed inadequately. However, a normal ECG reliably ruled out LVSD in about half of subjects (NPV 97%, proportion testing negative 46%) missing only 1 in 7 cases (sensitivity 85%; see Table 2). Previous MI was an unreliable marker, missing 3 of 4 cases of LVSD (sensitivity 24%) and 9 of 10 cases of HFpEF (sensitivity 12%).

### Test combinations

Combinations of ECG, clinical symptoms and signs, BNP and NT-proBNP and blood markers were investigated; there was no increase in the balance of diagnostic performance when various combinations were analysed. A range of test combinations were explored using ‘abnormal ECG’ as a starting point to reflect clinical guidelines. Individually, a natriuretic peptide test had a greater area under the curve than in combination with an abnormal ECG or other sign or symptom. ECG results did not increase diagnostic precision if added to a natriuretic peptide test and clinical assessment (see Table S1 for further details and test combinations).

### Incidental clinical findings

A high proportion of residents had test results outside the normal range which may have indicated conditions other than HF (see Table S2). For example, one quarter had raised creatinine; 51% had raised urea; 7% had a raised serum troponin. Other more definitive diagnoses included two residents whose study assessment resulted in an urgent referral for a permanent pacemaker, and one who needed reassessment for a substantially elevated blood pressure.

## Discussion

The study used consultant-interpreted portable echocardiography as a reference standard to diagnose HF due to LVSD and HFpEF in the older long-term care population. Against this reference a wide range of tests and clinical symptoms and signs were evaluated for the first time in this older institutionalised population, including the first formal evaluation of the diagnostic utility of several novel biomarkers. Diagnostic assessments (including echocardiogram and venepuncture) were feasible and acceptable.

BNP and NT-proBNP provided reasonable rule out tests for LVSD, reducing by about two-thirds the need for referral for echocardiographic assessment, and may appear cost-effective. However, by this route one in four patients would be missed, delaying diagnosis and effective treatment. Our findings indicate the limited utility of hs-CRP, copeptin, MP-proADM and MP-proANP in this population. There were no adequate diagnostic tests for HFpEF [32], which may be an important issue as consensus for treating HFpEF emerges. Thus sole use of individual biomarkers may not currently be sufficient for appropriate decisions about care pathways in this population [33].

For all tests, using lower test thresholds (enhancing ‘rule-in’) would reduce the number of missed cases of HF but result in unacceptably large numbers of patients falsely testing positive (thus requiring echocardiography) meaning that the utility of current tests is limited. Current NICE guidelines [9] recommend automatic referral for echocardiography for people with previous MI and natriuretic peptide screening for those without. The performance of BNP and NT-proBNP was marginally improved when limited to residents without previous MI (see Table 2).

### Study limitations

The level of heart failure in the study population [LVSD 34/399; HFpEF 57/399] was modest, and the possibility of missed heart failure cannot be completely ruled out even with echocardiography. Samples were derived from a prevalence study [22]; thus an increase in numbers was not feasible here. While this study has highlighted the need for differentiation in biomarker cut-offs for this population, larger scale studies are required to confirm the performance of these novel and routine biomarkers in this population.

There is wide variation in previously reported cut-off values for NT-proBNP and BNP for older people. Used as a rule-out test for LVSD, our findings suggest NT-proBNP and BNP cut-offs of 1000 pg/ml and 145 pg/ml respectively. These values differ from those reported by others studying similar populations, which at 93–450 pg/ml for NT-proBNP and from 40–100 pg/ml for BNP [1], [7], [8] are more similar to the thresholds in this study for HFpEF (477 pg/ml and 110 pg/ml respectively). It is possible that previous studies have included undifferentiated HF (LVSD and HFpEF), where there is no evidence of a differential diagnosis or separate cut-off values. The low prevalence of LVSD in this study (34/399) prevented sub-group evaluation of biomarker performance according to gender, age or disease severity. However, rates of HFpEF were higher than anticipated (57/399). A similar overall prevalence of HF in our findings and those of others may reflect a lack of historical differentiation between HFpEF from LVSD. The high incidence of HFpEF detected suggests the need for further research in this population, in order to establish clear treatment guidelines [34].

Participant recruitment was challenging due to organisational barriers as well as residents' physical and cognitive limitations. Nonetheless one third of those approached participated. While baseline demographics of participants and non-participants were similar it is possible that non-participation occurred, in part, as a result of a higher burden of ill-health. We recognise that screening asymptomatic patients does not reflect routine clinical assessment, where doctors may attend on the basis of symptoms suggesting HF or other disease. On-site assessment using echocardiography was acceptable and feasible to older people in care but remains a time and resource-intensive option. A simple, definitive test for HF in this population is elusive. Until such a test emerges, echocardiography remains feasible and acceptable to residents, and home-based access would offer optimal diagnosis when access to other service configurations may be problematic.

### Conclusion

No biomarker test, individually or in combination, adequately balanced case finding and rule-out for heart failure in this population; currently, in-situ echocardiography provides the only adequate diagnostic assessment. Commissioners and policy makers should consider the routine provision of on-site portable echocardiography, particularly if, in addition to LVSD, consensus emerges about the appropriate treatment of HFpEF.

## Supporting Information

Table S1 Diagnostic test performance (area under the curve) of combinations of diagnostic markers, signs and symptoms in detecting LVSD.
(DOCX)

Table S2 Findings of tests not specific to heart failure.
(DOCX)
