# Supplementary material for: Utility of Biomarkers in the Differential Diagnosis of Heart Failure in Older People: Findings from the Heart Failure in Care Homes (HFinCH) Diagnostic Accuracy Study
Source: PLoS One. 2013 Jan 11;8(1):e53560. doi: 10.1371/journal.pone.0053560 (PMC3543443; doi:10.1371/journal.pone.0053560)
Supplement: Table S1 — Diagnostic test performance (area under the curve) of combinations of diagnostic markers, signs and symptoms in detecting LVSD. (DOCX) [file pone.0053560.s001.docx]

**Table S1:** Diagnostic test performance (area under the curve) of combinations of
diagnostic markers, signs and symptoms in detecting LVSD.

| **Combination of markers, signs and symptoms** | **Area under the Curve** | **95% confidence interval** |
| --- | --- | --- |
| Abnormal ECG AND BNP>=140 | 0.72 | 0.62 - 0.82 |
| Abnormal ECG AND BNP>=140 AND abnormal JVP | 0.66 | 0.55 - 0.77 |
| Abnormal ECG AND BNP>=140 AND lung crackles | 0.54 | 0.43 - 0.64 |
| Abnormal ECG AND abnormal JVP | 0.62 | 0.52 - 0.72 |
| Abnormal ECG AND lung crackles | 0.60 | 0.48 - 0.71 |
| Abnormal ECG AND orthopnea (sitting OR standing) | 0.57 | 0.46 - 0.68 |
| Abnormal ECG AND oedema | 0.54 | 0.43 - 0.65 |
| Abnormal ECG AND systolic BP (>=140) | 0.56 | 0.45 - 0.67 |
| Abnormal ECG AND diastolic BP (>=90) | 0.50 | 0.40 - 0.60 |
| Abnormal ECG AND heart rate>=100 | 0.51 | 0.40 - 0.61 |
| Abnormal ECG AND respiratory rate >=20 | 0.60 | 0.49 - 0.71 |
| Abnormal ECG AND NTproBNP>=1030 | 0.72 | 0.62 - 0.82 |
| Abnormal ECG AND NTproBNP (>=1030) AND BNP (>=140) | 0.74 | 0.63 - 0.84 |
| Abnormal ECG AND NTproBNP (>=1030) AND JVP | 0.66 | 0.55 - 0.76 |
| Abnormal ECG AND NTproBNP (>=1030) AND lung crackles | 0.61 | 0.50 - 0.72 |
| Abnormal ECG AND NTproBNP (>=1030) AND orthopnea (sitting OR standing) | 0.57 | 0.46 - 0.68 |
| Abnormal ECG AND NTproBNP (>=1030) AND oedema | 0.57 | 0.46 - 0.68 |
| Abnormal ECG AND NTproBNP (>=1030) AND systolic BP (>=140) | 0.57 | 0.45 - 0.68 |
| Abnormal ECG AND NTproBNP (>=1030) AND diastolic BP (>=90) | 0.50 | 0.39 - 0.60 |
| Abnormal ECG AND NTproBNP (>=1030) AND pulse (>=100) | 0.51 | 0.41 - 0.62 |
| Abnormal ECG AND NTproBNP (>=1030) AND respiratory rate (>=20) | 0.59 | 0.48 - 0.70 |
