# Supplementary material for: Utility of Biomarkers in the Differential Diagnosis of Heart Failure in Older People: Findings from the Heart Failure in Care Homes (HFinCH) Diagnostic Accuracy Study
Source: PLoS One. 2013 Jan 11;8(1):e53560. doi: 10.1371/journal.pone.0053560 (PMC3543443; doi:10.1371/journal.pone.0053560)
Supplement: Table S2 — Findings of tests not specific to heart failure. (DOCX) [file pone.0053560.s002.docx]

**Table S2:** Findings of tests not specific to heart failure

| **Blood** | **No of** | **Normal** | **Outside range** | | **No HF (N=308)** | | |  | **Undifferentiated HF (n=91)** | | | | |  | **LVSD (n=34)** | | | | |  | **HFPEF (n=57)** | | | | |
| --- | --- | --- | --- | --- | --- | --- | --- | --- | --- | --- | --- | --- | --- | --- | --- | --- | --- | --- | --- | --- | --- | --- | --- | --- | --- |
| **test** | **residents** | **range** | **N** | **%** | **Mean** | **Range** | **SD** |  | **Mean** | **Range** | **SD** | **AUC** | **(95% CI)** |  | **Mean** | **Range** | **SD** | **AUC** | **(95% CI)** |  | **Mean** | **Range** | **SD** | **AUC** | **(95% CI)** |
| Na+ | 395 | 135-145 mmol/l | 46 | 12 | 140 | 129-148 | 3 |  | 140 | 126-148 | 4 | 0.5 | (0.43-0.58) |  | 141 | 126-148 | 5 | 0.57 | (0.46-0.68) |  | 140 | 129-147 | 4 | 0.45 | (0.36-0.53) |
| K+ | 384 | 3.5-5.0mmol/l | 26 | 7 | 4 | 3-44 | 2 |  | 4.3 | 2.8-5.7 | 0.5 | 0.53 | (0.46-0.61) |  | 4 | 3-6 | 0.4 | 0.51 | (0.41-0.61) |  | 4 | 3-6 | 0.5 | 0.52 | (0.43-0.61) |
| Urea | 395 | 2.5-7.0mmol/l | 202 | 51 | 8 | 3-29 | 4 |  | 8.5 | 2.9-18 | 3 | 0.64 | (0.57-0.70) |  | 8 | 4-18 | 3 | 0.54 | (0.44-0.64) |  | 9 | 3-15 | 3 | 0.64 | (0.57-0.72) |
| Creatinine | 395 | 50-110µmol/l | 93 | 24 | 91 | 35-484 | 40 |  | 102 | 38-311 | 41 | 0.61 | (0.54-0.68) |  | 108 | 48-311 | 50 | 0.62 | (0.52-0.71) |  | 99 | 38-235 | 35 | 0.58 | (0.50-0.67) |
| Chloride | 395 | 96-106mmol/l | 130 | 33 | 104 | 48-114 | 6 |  | 103 | 85-116 | 5.2 | 0.48 | (0.40-0.55) |  | 104 | 85-114 | 6 | 0.54 | (0.43-0.66) |  | 103 | 90-116 | 5 | 0.44 | (0.36-0.52) |
| Hb | 391 | 12.0-16.0g/dl | 173 | 44 | 12 | 6.2-18 | 2 |  | 12 | 6.2-16 | 1.7 | 0.34 | (0.27-0.41) |  | 12 | 9-16 | 2 | 0.47 | (0.36-0.57) |  | 11 | 6-15 | 2 | 0.31 | (0.24-0.38) |
| WCC | 391 | 4.0-11.0x10-6g/l | 35 | 9 | 7 | 3-19 | 2 |  | 8.4 | 3.5-140 | 14 | 0.52 | (0.45-0.58) |  | 7 | 4-15 | 2 | 0.48 | (0.38-0.57) |  | 9 | 4-140 | 18 | 0.53 | (0.45-0.61) |
| MCV | 391 | 82-98fl | 89 | 23 | 92 | 71-123 | 7 |  | 92 | 75-119 | 7.9 | 0.48 | (0.40-0.55) |  | 91 | 75-106 | 7 | 0.48 | (0.37-0.60) |  | 92 | 76-119 | 8 | 0.51 | (0.43-0.60) |
| Platelets | 391 | 150-450x10-6g/l | 30 | 8 | 275 | 45-3741 | 224 |  | 251 | 14-662 | 88 | 0.47 | (0.40-0.54) |  | 236 | 110-662 | 93 | 0.39 | (0.30-0.47) |  | 259 | 14-441 | 85 | 0.53 | (0.44-0.61) |
| Haematocrit | 391 | 0.36-0.44% | 169 | 43 | 0.4 | 0.3-0.5 | 0.05 |  | 0.36 | 0.03-0.5 | 0.06 | 0.36 | (0.29-0.43) |  | 0.4 | 0.3-0.5 | 0.05 | 0.50 | (0.39-0.60) |  | 0.4 | 0.03-0.5 | 0.06 | 0.32 | (0.25-0.40) |
| Bilirubin | 392 | 0-17µmol/l | 13 | 3 | 8 | 2-31 | 4 |  | 7.6 | 3-22 | 3.3 | 0.49 | (0.42-0.56) |  | 9 | 3-22 | 4 | 0.62 | (0.52-0.72) |  | 7 | 3-13 | 2 | 0.41 | (0.34-0.49) |
| Albumin | 395 | 34-50g/l | 20 | 5 | 40 | 4-48 | 4 |  | 39 | 12-46 | 4.5 | 0.48 | (0.41-0.56) |  | 39 | 33-46 | 3 | 0.47 | (0.36-0.57) |  | 39 | 12-46 | 5 | 0.49 | (0.41-0.57) |
| Total protein | 392 | 60-80g/l | 34 | 9 | 68 | 52-85 | 6 |  | 68 | 50-88 | 6.2 | 0.47 | (0.40-0.54) |  | 68 | 55-81 | 6 | 0.51 | (0.40-0.61) |  | 67 | 50-88 | 6 | 0.45 | (0.37-0.53) |
| ALT | 392 | <40iu/l | 6 | 2 | 17 | 4-130 | 10 |  | 16 | 5-116 | 12.4 | 0.45 | (0.38-0.53) |  | 15 | 5-28 | 5 | 0.49 | (0.39-0.60) |  | 17 | 6-116 | 15 | 0.43 | (0.35-0.52) |
| ALP | 394 | M: 40-129iu/l | 94 | 32 | 102 | 42-478 | 53 |  | 106 | 33-485 | 62 | 0.53 | (0.46-0.60) |  | 103 | 42-251 | 42 | 0.52 | (0.42-0.62) |  | 108 | 33-485 | 71 | 0.51 | (0.43-0.60) |
|  |  | F 35-104iu/l | 16 | 5 |  |  |  |  |  |  |  |  |  |  |  |  |  |  |  |  |  |  |  |  |  |
| Gamma GT | 383 | 0-38u/l | 90 | 24 | 36 | 5-422 | 51 |  | 43 | 3-285 | 55 | 0.53 | (0.46-0.60) |  | 56 | 5-285 | 68 | 0.60 | (0.50-0.70) |  | 36 | 3-264 | 45 | 0.49 | (0.41-0.57) |
| TSH | 387 | 0.35-5.5miu/l | 52 | 13 | 3 | 0.05-62 | 5 |  | 2.4 | 0.06-9.3 | 2 | 0.51 | (0.44-0.59) |  | 2 | 0.06-9 | 2 | 0.48 | (0.36-0.59) |  | 2 | 0.1-9 | 2 | 0.52 | (0.44-0.61) |
| Cholesterol | 393 | <5mmol/l | 138 | 35 | 5 | 2-9 | 1 |  | 4.4 | 2.4-6.9 | 1.1 | 0.42 | (0.35-0.50) |  | 4 | 2-6 | 1 | 0.39 | (0.28-0.50) |  | 5 | 3-7 | 1 | 0.47 | (0.39-0.55) |
| Triglycerides | 393 | <1.8mmol/l | 96 | 25 | 2 | 0.5-8 | 0.8 |  | 1.6 | 0.4-6.9 | 0.9 | 0.53 | (0.46-0.60) |  | 2 | 1-7 | 1 | 0.50 | (0.41-0.60) |  | 2 | 0.4-4 | 0.7 | 0.55 | (0.46-0.63) |
| HDL | 390 | M: >0.9mmol/l | 26 | 1 | 2 | 0.4-3 | 0.4 |  | 1.4 | 0.7-3.1 | 0.4 | 0.44 | (0.38-0.51) |  | 1 | 1-2 | 0.3 | 0.38 | (0.29-0.48) |  | 2 | 0.7-3 | 0.4 | 0.51 | (0.43-0.59) |
|  |  | F >1.1mmol/l | 92 | 90 |  |  |  |  |  |  |  |  |  |  |  |  |  |  |  |  |  |  |  |  |  |
| LDL | 390 | <3mmol/l | 105 | 27 | 3 | 0.8-7 | 1 |  | 2.3 | 0.8-4.3 | 0.9 | 0.43 | (0.35-0.50) |  | 2 | 1-4 | 1 | 0.42 | (0.31-0.53) |  | 2 | 1-4 | 1 | 0.44 | (0.36-0.53) |
| Troponin I | 394 | <0.06ug/l | 27 | 7 | 0.09 | 0.02-20 | 1 |  | 0.1 | 0.01-5.3 | 0.56 | 0.58 | (0.50-0.65) |  | 0.20 | 0.03-5 | 1 | 0.64 | (0.53-0.75) |  | 0.04 | 0.01-0.2 | 0.02 | 0.53 | (0.44-0.61) |
| Glucose | 393 | 3.0-6.0mmol/l | 249 | 63 | 7 | 3-21 | 3 |  | 7.5 | 3.6-22 | 3.2 | 0.50 | (0.43-0.57) |  | 8 | 4-20 | 3 | 0.57 | (0.48-0.67) |  | 7 | 4-22 | 3 | 0.46 | (0.38-0.55) |
| Serum Calcium | 394 | 2.2-2.6mmol/l | 153 | 39 | 2 | 2-3 | 0.1 |  | 2.2 | 1.9-2.5 | 0.1 | 0.47 | (0.40-0.55) |  | 2 | 2-3 | 0.1 | 0.47 | (0.37-0.56) |  | 2 | 2-3 | 0.1 | 0.48 | (0.39-0.57) |
| Corrected Calcium | 394 | 2.15-2.6mmol/l | 73 | 19 | 2 | 2-3 | 0.1 |  | 2.2 | 1.9-2.5 | 0.1 | 0.48 | (0.40-0.55) |  | 2 | 2-3 | 0.1 | 0.47 | (0.38-0.56) |  | 2 | 2-3 | 0.1 | 0.48 | (0.39-0.57) |
| Na+ | 395 | 135-145 mmol/l | 46 | 12 | 140 | 129-148 | 3 |  | 140 | 126-148 | 4 | 0.5 | (0.43-0.58) |  | 141 | 126-148 | 5 | 0.57 | (0.46-0.68) |  | 140 | 129-147 | 4 | 0.45 | (0.36-0.53) |
